# Cancer Mortality in an Ageing Population: Evidence of Sex-Specific Divergence from a National Study in Poland

**DOI:** 10.3390/cancers18030447

**Published:** 2026-01-30

**Authors:** Monika Burzyńska, Małgorzata Pikala

**Affiliations:** 1 Department of Epidemiology and Biostatistics, Public Health Observatory, Medical University of Lodz, 90-752 Lodz, Poland; malgorzata.pikala@umed.lodz.pl; 2 Department of Internal Medicine and Geriatric Cardiology, Centre of Postgraduate Medical Education, 01-813 Warszawa, Poland

**Keywords:** malignant neoplasms, ageing, geriatric oncology, epidemiology, mortality trends

## Abstract

**Background**: Malignant neoplasms are a leading cause of death among older adults, and population ageing is expected to further increase the cancer burden. Analysing long-term mortality trends is essential for evaluating the effectiveness of cancer prevention and oncological care. The aim of the study was to analyse long-term trends in mortality from malignant neoplasms among adults aged 65 years and older in Poland between 2000 and 2022. **Methods**: This nationwide population-based study analysed all deaths due to malignant neoplasms among Polish residents aged ≥65 years between 2000 and 2022 using national mortality data from Statistics Poland. Mortality trends were assessed separately for women and men in early (65–74 years) and late (≥75 years) old age. Standardised death rates (SDRs) were calculated using the European Standard Population. Joinpoint regression was applied to estimate annual (APC) and average annual percentage changes (AAPC). **Results:** Overall cancer mortality declined steadily among men in both age groups, while trends among women were heterogeneous. In men aged 65–74 years, SDRs decreased from 1140.1 to 1006.0 per 100,000 (AAPC = −1.7%), largely driven by declining lung cancer mortality (AAPC = −2.6%); similar patterns were observed in men aged ≥75 years. Among women aged 65–74 years, overall cancer mortality declined modestly (AAPC = −0.4%), but lung cancer mortality increased markedly, with SDRs nearly doubling between 2000 and 2022 (AAPC = 3.5%). **Conclusions**: Sustained declines among men contrast with less favourable trends among women, particularly for smoking-related cancers, highlighting the need for targeted prevention, screening, and age-adapted oncological care.

## Simple Summary

Population ageing is leading to a growing burden of cancer among older adults, making it increasingly important to understand long-term mortality patterns in this age group. In this study, we analysed changes in cancer mortality among people aged 65 years and older in Poland over a 23-year period. We examined differences by sex, age group, and cancer type to identify which malignancies contribute most to mortality in early and late old age. Our findings show that cancer mortality has declined among older men but remains more variable among women, with rising deaths from lung and breast cancer in the oldest age groups. These trends reflect past differences in smoking behaviour, cancer prevention, and access to early diagnosis and treatment. By highlighting sex- and age-specific patterns, this study provides evidence to support more targeted cancer prevention, screening, and care strategies for ageing populations.

## 1. Introduction

In the context of rapid global population ageing, the burden of malignant neoplasms is expected to intensify substantially. According to projections from the World Health Organization, by 2030 one in six individuals worldwide will be aged 60 years or older, and by 2050 this population will reach 2.1 billion, with approximately 80% residing in low- and middle-income countries. As cancer incidence increases markedly with age, these demographic shifts are likely to translate into a considerable rise in cancer-related morbidity and mortality among older adults [1].

Population ageing is accompanied not only by a growing prevalence of chronic diseases but also by profound changes in the structure and dynamics of mortality causes. Malignant neoplasms represent a particularly important public health challenge in older age due to their multifactorial aetiology, long latency periods, and strong associations with cumulative lifetime exposures, including tobacco use, diet, occupational hazards, and environmental factors. In addition, age-related physiological changes, multimorbidity, and variations in access to preventive and therapeutic services may significantly influence cancer outcomes in older populations [2,3,4].

From a public health and clinical perspective, the analysis of long-term cancer mortality trends stratified by sex and age provides valuable insight into the effectiveness of prevention strategies, shifts in exposure to major risk factors, and the impact of advances in early detection and oncological care. Such analyses also help to identify emerging disparities in cancer burden and to inform more targeted, age-adapted prevention and healthcare planning in ageing societies [5,6].

Without effective and age-sensitive interventions—encompassing primary prevention, widespread participation in screening programmes, timely diagnosis, and appropriate oncological management—the burden of malignant neoplasms in older adults is likely to continue to increase. Strengthening cancer prevention policies, improving early detection, and integrating geriatric oncology principles into health systems will therefore be essential to mitigate the future impact of cancer in ageing populations [7].

In Poland, cancer mortality trends in the general population have been systematically monitored for decades through nationwide data collected by the National Cancer Registry [8]. These data indicate that cancer remains one of the leading causes of death and its burden is strongly shaped by demographic ageing. Despite the availability of this data, evidence on detailed cancer mortality long-term trends assessment using advanced statistical methods, specifically among individuals aged 65 years and older in Poland, remains limited. Addressing this gap is essential for a better understanding of cancer burden in ageing societies and for informing targeted public health strategies.

Therefore, the aim of this study was to analyse long-term trends in mortality from malignant neoplasms among adults aged 65 years and older in Poland between 2000 and 2022, with particular emphasis on sex- and age-specific differences and on the most common cancer sites contributing to mortality in early and late old age.

## 2. Materials and Methods

The analysis was based on a nationwide mortality database comprising all registered deaths among residents of Poland aged 65 years and older in the years 2000–2022, obtained from Statistics Poland—the official national statistical office responsible for the collection, processing, and dissemination of demographic and health statistics in Poland. Cause of death information is derived from death certificates coded according to the International Classification of Diseases (ICD-10). In total, 6,645,408 deaths were included in the statistical analyses. Within this dataset, deaths attributed to malignant neoplasms (ICD-10 codes C00–C97) were extracted, with particular emphasis on the most prevalent cancer sites in older adults: malignant neoplasms of the bronchus and lung (C34), stomach (C16), colorectum (C18–C20), breast (C50), prostate (C61), and pancreas (C25).

Given the well-recognised differences in morbidity and mortality patterns by sex and age in older populations, all analyses were conducted separately for women and men and stratified into two age categories: 65–74 years (early old age) and 75 years and older (late old age).

To examine changes in the distribution of causes of death over time, proportional mortality ratios were calculated for the initial and final years of the study period. In addition, age-standardised death rates (SDRs) were estimated using the direct standardisation method based on the 2012 European Standard Population [9]. Temporal trends in SDRs were assessed using joinpoint regression analysis implemented in the Joinpoint Regression Program developed by the U.S. National Cancer Institute within the Surveillance, Epidemiology and End Results (SEER) framework [10]. This method is an advanced version of linear regression, where the time trend is expressed with a broken line, which is a sequence of segments joined in joinpoints. In these points, the change in the value is statistically significant (*p* < 0.05). For each identified time segment, annual percentage changes (APC) were calculated, along with the average annual percentage change (AAPC) for the entire study period, accompanied by 95% confidence intervals. Poland is administratively divided into 16 voivodeships (NUTS 2 regions); therefore, the analysis additionally included a territorial comparison of standardised death rates (SDRs) for malignant neoplasms across voivodeships in 2022.

The study protocol received approval from the Bioethics Committee of the Medical University of Lodz (approval No. RNN/422/12/KB). In accordance with national regulations and institutional policies, written informed consent was not required due to the use of anonymised registry data.

## 3. Results

Changes in mortality rates due to malignant neoplasms differed by sex. Among men in both early and late old age, mortality rates declined, whereas among women periodic decreases and increases were observed in both age groups (Supplementary Tables S1 and S2).

**Figure 1:**
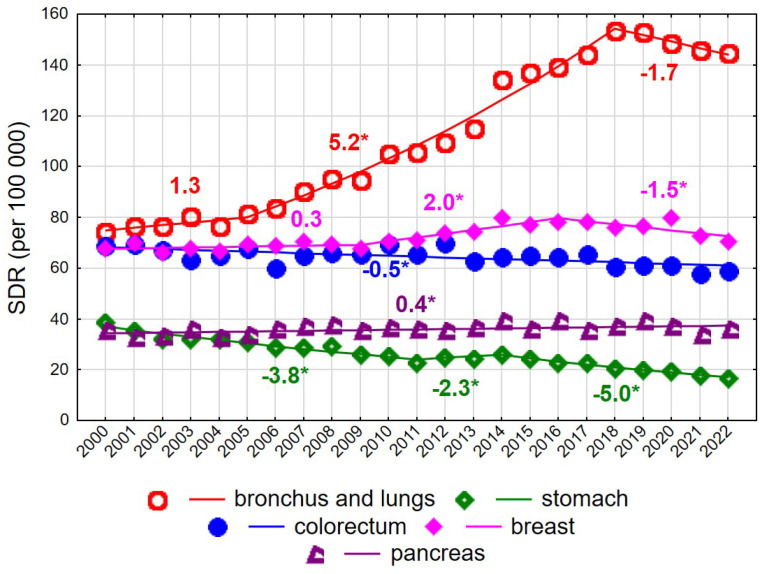
Mortality trends due to the most common causes of death in the class of malignant tumours in the years 2000–2022 among women aged 65–74 years. * *p* < 0.05.

**Figure 2:**
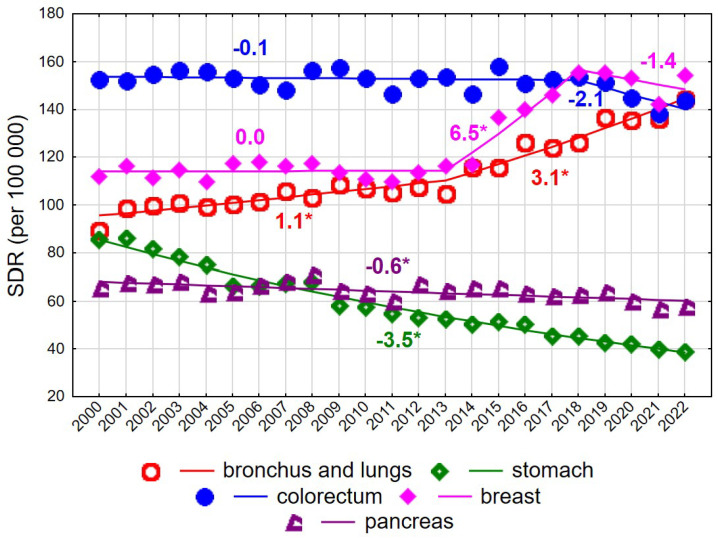
Mortality trends due to the most common causes of death in the class of malignant tumours in the years 2000–2022 among women aged 75+. * *p* < 0.05.

Among women aged 65–74 years, a decline in SDR values was recorded over the entire observation period 2000–2022, from 631.4 to 580.9 (AAPC = −0.4%, *p* < 0.05). However, the trend changed direction twice. Following a decrease between 2000 and 2006 at an annual rate of −1.0% (*p* < 0.05), SDRs increased at a rate of 0.5% per year between 2006 and 2019 (*p* < 0.05), followed by a renewed decline after 2019 at a rate of −2.9% annually (*p* < 0.05). Unfavourable changes in mortality trends among women aged 65–74 years were driven primarily by mortality from malignant neoplasms of the bronchus and lung. For this cause of death, a slight and statistically non-significant increase in SDRs was observed in 2000–2005, followed by a rapid and statistically significant increase in 2005–2018 (APC = 5.2%, *p* < 0.05), and a non-significant decline after 2018. Over the entire 23-year period, the AAPC amounted to 3.5% (*p* < 0.05), resulting in an increase in SDRs from 74.2 in 2000 to 144.6 in 2022 (Supplementary Tables S1 and S3). Periodic increases and decreases were also observed for mortality due to malignant neoplasms of the breast. In 2000–2009, a statistically non-significant increase was noted, followed by a statistically significant rise in 2009–2016 at an annual rate of 2.0%, and a statistically significant decline after 2016 at a rate of −1.5%. As a result, the SDR for breast cancer mortality in women aged 65–74 years changed from 67.8 in 2000 to 70.5 in 2022 (AAPC = 0.3%, *p* > 0.05). A small but statistically significant increase was observed for mortality due to malignant neoplasms of the pancreas (APC = 0.4%, *p* < 0.05). In contrast, a steady decline was noted for mortality due to malignant neoplasms of the stomach (AAPC = −3.5%, *p* < 0.05) and the colorectum (APC = −0.4%, *p* < 0.05). Among women aged 75 years and older, changes in overall cancer mortality rates were not statistically significant (AAPC = −0.4%, *p* > 0.05) (Figure 1 and Figure 2, Supplementary Tables S1 and S3).

Up to 2017, the highest SDR values among women aged ≥75 years were observed for colorectal cancer (Figure 2). The SDR for this cause was 152.5 in 2000 and 143.7 in 2022, with the overall decline being statistically non-significant. Rapidly increasing breast cancer mortality rates between 2013 and 2018 (APC = 6.5% annually) resulted in breast cancer becoming the leading cause of cancer death in women of late old age, reaching an SDR of 154.1 per 100,000 in 2022. A steady increase was also observed for mortality due to malignant neoplasms of the bronchus and lung (AAPC = 1.9%, *p* < 0.05), with an acceleration after 2013 (APC = 1.1% in 2000–2013 and 3.1% in 2013–2022, both *p* < 0.05). Consequently, the SDR increased from 89.4 in 2000 to 144.3 in 2022. In contrast, steady declines were observed for mortality due to malignant neoplasms of the stomach (AAPC = −3.5%, *p* < 0.05) and pancreas (AAPC = −0.6%, *p* < 0.05) (Figure 2, Supplementary Tables S1 and S3).

**Figure 3:**
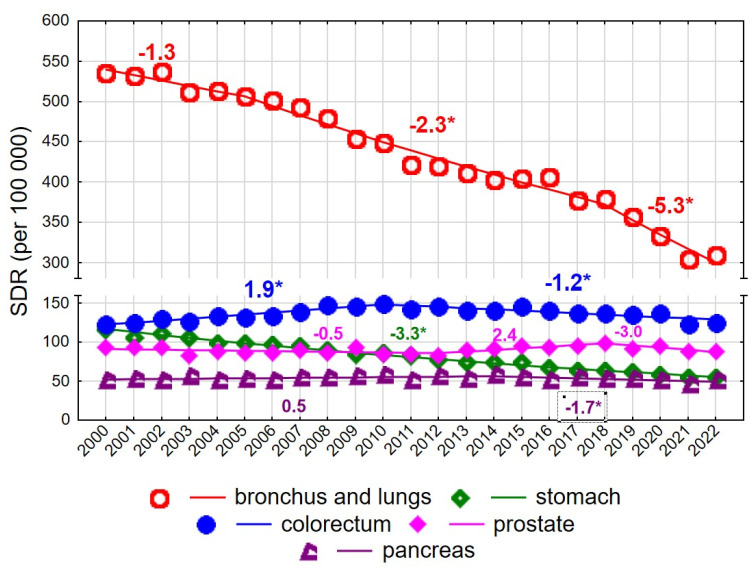
Mortality trends due to the most common causes of death in the class of malignant tumours in the years 2000–2022 among men aged 65–74 years. * *p* < 0.05.

Among men aged 65–74 years, the SDR for malignant neoplasms decreased from 1140.1 in 2000 to 1006.0 in 2022 (AAPC = −1.7%, *p* < 0.05) (Figure 3, Supplementary Tables S1 and S2).

The rate of decline changed twice: following a statistically non-significant decrease in 2000–2004, a significant decline occurred in 2004–2018 (APC = −1.5%, *p* < 0.05), which accelerated in 2018–2022 (APC = −5.3%, *p* < 0.05). A particularly rapid decline was observed for mortality due to malignant neoplasms of the bronchus and lung (AAPC = −2.6%, *p* < 0.05) (Figure 3). Despite this decline, lung cancer remained the leading cause of cancer death among men aged 65–74 years, with the SDR decreasing from 535.9 in 2000 to 308.7 in 2022. The pace of decline changed over time: it was non-significant in 2000–2005, −2.3% annually in 2005–2018, and −5.3% in 2018–2022 (both *p* < 0.05).

Colorectal cancer was the second most common cause of cancer death in men aged 65–74 years, with an increase observed in 2000–2010 (APC = 1.9%, *p* < 0.05), followed by a decline in 2010–2022 (APC = −1.2%, *p* < 0.05). No statistically significant changes were observed for mortality due to malignant neoplasms of the prostate in this age group; the SDR was 93.6 in 2000 and 87.9 in 2022 (AAPC = −0.2%, *p* > 0.05). In contrast, mortality due to malignant neoplasms of the stomach declined significantly at a constant annual rate of −3.3%, resulting in a decrease in the SDR from 116.5 in 2000 to 54.3 in 2022. Mortality due to malignant neoplasms of the pancreas also declined after 2014 (APC = −1.7%, *p* < 0.05).

**Figure 4:**
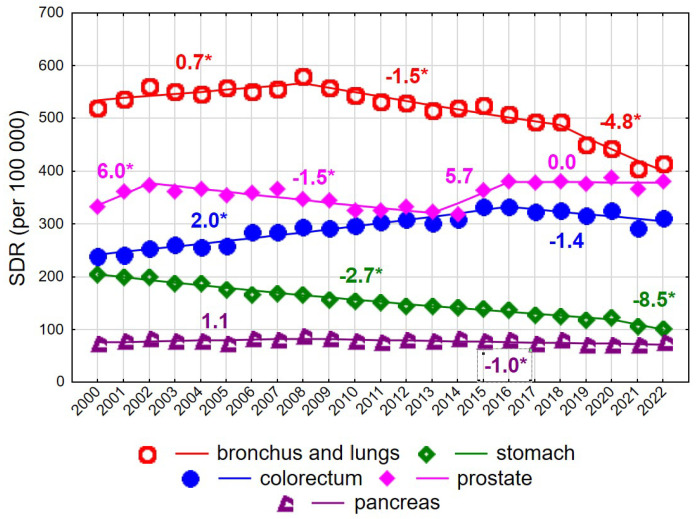
Mortality trends due to the most common causes of death in the class of malignant tumours in the years 2000–2022 among men aged 75+. * *p* < 0.05.

Among men aged ≥75 years, mortality rates due to malignant neoplasms declined steadily at an annual rate of −0.5% (*p* < 0.05), from 2226.5 in 2000 to 2076.9 in 2022 (Supplementary Tables S2 and S3). As in younger men, this decline was largely driven by decreasing mortality due to malignant neoplasms of the bronchus and lung after 2008 (Figure 4). The annual rate of decline was −1.5% in 2008–2018 (*p* < 0.05) and accelerated to −4.8% in 2018–2022 (*p* < 0.05), resulting in a decrease in the SDR from 580.6 in 2008 to 414.8 in 2022. Prostate cancer constituted the second leading cause of cancer death in men aged ≥75 years. SDR values showed periodic increases and decreases, with statistically significant changes observed only in 2002–2013 (APC = −1.5%, *p* < 0.05). In 2022, the SDR for prostate cancer reached 381.7, approaching the SDR for lung cancer.

Mortality due to colorectal cancer, the third leading cause of cancer death in men aged ≥75 years, increased in 2000–2016 at an annual rate of 2.0% (*p* < 0.05), followed by a statistically non-significant decline. A rapid and statistically significant decrease was observed for mortality due to malignant neoplasms of the stomach, with an annual decline of −2.7% in 2000–2020 (*p* < 0.05), accelerating to −8.5% in 2020–2022 (*p* < 0.05). As a result, SDR values decreased more than twofold between 2000 and 2022, from 205.3 to 101.7. Mortality due to malignant neoplasms of the pancreas also declined after 2008 (APC = −1.0%, *p* < 0.05).

Substantial territorial variation in SDRs for mortality due to malignant neoplasms was observed across Poland. The lowest SDRs for all cancers combined, in both analysed age groups and in both men and women, were found in the Podkarpackie voivodeship (Supplementary Tables S4 and S5). The highest SDRs among individuals aged 65–74 years, in both sexes, were observed in the Pomorskie voivodeship, whereas among those aged 75 years and older, the highest SDRs occurred in the Małopolskie voivodeship. In the Pomorskie voivodeship, the SDR for women in early old age was 675.2 and was 1.5 times higher than in the Podkarpackie voivodeship (441.6). Among men, the corresponding ratio was 1.3 (1127.0 vs. 875.0). In the late old-age group, the SDR for women in the Małopolskie voivodeship was 1162.9 and was 1.4 times higher than in the Podkarpackie voivodeship (829.1). Among men, the SDR ratio between the Małopolskie and Podkarpackie voivodeships was also 1.4 (2287.9 vs. 1627.2). The Podkarpackie voivodeship consistently showed the lowest SDRs for malignant neoplasm of the bronchus and lung across all four analysed sex- and age-specific subgroups. Additionally, it had the lowest SDRs for malignant neoplasm of the colorectum among men and women aged 75 years and older, and for breast cancer among women aged 75 years and older. The largest interregional differences, defined as SDR ratios exceeding 2.0 between voivodeships with the highest and lowest values, were observed for stomach cancer among women in late old age (ratio 3.3), lung cancer among women in late old age (2.8) and early old age (2.2), stomach cancer among women in early old age (2.5), and pancreatic cancer among women in early old age (2.3) and men in late old age (2.2).

## 4. Discussion

The present study revealed distinct sex- and age-specific patterns in mortality due to malignant neoplasms among older adults in Poland between 2000 and 2022. Overall, declining mortality trends were observed among men in both early and late old age, whereas among women mortality patterns were more heterogeneous, with periods of both increase and decline depending on age group and cancer site. These findings reflect the combined influence of long-term changes in risk factor exposure, demographic ageing, healthcare system performance, and cohort effects.

A key finding of this study is the pronounced decline in standardised death rates (SDRs) from lung cancer among men. This trend is consistent with evidence from other countries and can be attributed primarily to the substantial reduction in smoking prevalence among male populations. Although smoking prevalence has declined in both sexes, smoking cessation occurred earlier and more extensively among men, leading to earlier and more pronounced reductions in lung cancer mortality. The observed decline is also likely influenced by broader lifestyle changes, including healthier dietary patterns, reduced alcohol consumption, and decreased occupational exposure to carcinogenic agents [11,12,13].

The decrease in lung cancer mortality among men is closely linked to reductions in tobacco consumption initiated in the 1980s and 1990s. Numerous studies indicate that such trends were not observed to the same extent among women [14]. In several countries, including the United Kingdom and the United States, smoking prevalence among women increased during or shortly after World War II, while in other regions it rose later, particularly during the 1970s. In addition to behavioural changes, improved early detection and advances in oncological treatment have contributed to declining lung cancer mortality among men. Projections suggest that lung cancer mortality rates may continue to decrease, potentially by as much as 79% by 2065 compared with 2015 levels [15]. However, these favourable trends appear less pronounced in Eastern Europe, possibly reflecting slower implementation of effective therapies and lower adherence to healthy lifestyle behaviours [16]. In Poland, according to the report *Malignant Neoplasms in Poland in 2021*, a declining trend in lung cancer incidence among men has been observed for approximately 15 years [17].

In contrast, the increase in lung cancer mortality among women observed in the present study aligns with findings from other investigations. Analyses conducted in several European Union countries and the United Kingdom indicate that lung cancer mortality among women aged 65 years and older is likely to continue rising in the coming years. One study projected increases of nearly 14% in France, 5.6% in Italy, and 5% in Spain. Importantly, the authors of that study did not assess the impact of the COVID-19 pandemic, which may further influence mortality trends through delays in oncological diagnosis and treatment [18]. Beyond reductions in smoking prevalence, additional decreases in lung cancer mortality may be achieved through improvements in early detection and treatment, particularly via the implementation of low-dose computed tomography (LDCT) screening. Evidence suggests that LDCT screening can reduce lung cancer mortality by approximately 20% compared with standard chest radiography [19].

The COVID-19 pandemic constitutes an important contextual factor for the interpretation of recent cancer mortality trends; however, its impact on mortality appears to be more complex than initially anticipated. In the present study, no increase in cancer mortality was observed during the pandemic period. On the contrary, the total number of deaths due to malignant neoplasms was lower than expected based on pre-pandemic trends. Between 2010 and 2019, cancer mortality in Poland demonstrated a statistically significant increasing trend (APC 1.1%), which would have resulted in an estimated 103,142 deaths during the subsequent period. Instead, 99,867 cancer-related deaths were observed, corresponding to 3275 fewer deaths than expected, consistently across both sexes [20]. These findings help explain why the mortality trend plots do not demonstrate a clear pandemic-related surge.

Several mechanisms may underlie this observation. First, cancer mortality reflects long disease trajectories, and short-term disruptions in healthcare delivery may not immediately translate into increased mortality. Second, underdiagnosis during the pandemic—resulting from reduced access to screening, diagnostic procedures, and specialist care—may have led to a temporary decrease in recorded cancer deaths, particularly for cancers with longer survival. Third, the adverse effects of delayed diagnosis and treatment are likely to manifest with a time lag and may become apparent in mortality statistics in subsequent years rather than during the acute pandemic period [19,21,22].

Previous reports and modelling studies from other countries have suggested potential increases in cancer mortality attributable to diagnostic and treatment delays during the COVID-19 pandemic. In several settings, cancer screening programmes were temporarily suspended or operated at reduced capacity. For example, in the United States, approximately 50% of centres interrupted screening programmes for breast, cervical, and colorectal cancer, while in Canada substantial reductions in screening activity were also reported [23,24]. Although these data indicate major disruptions in oncological care, the absence of an immediate increase in cancer mortality observed in our study suggests that the pandemic’s impact on mortality in Poland may be delayed rather than absent.

In Poland, screening activity declined substantially during the pandemic, with a 27% reduction in mammography and a 39% reduction in cervical cytology compared with 2019 levels. Reductions were also observed in primary healthcare visits, outpatient specialist care, and hospital treatment, including fewer referrals for surgical procedures and reduced use of radiotherapy and chemotherapy [25]. Continued monitoring of post-pandemic mortality trends will therefore be essential to fully assess the long-term consequences of healthcare disruptions on cancer outcomes.

The contrasting mortality trends observed between men and women underscore the importance of adopting a sex-specific perspective in cancer epidemiology among older adults [26]. While declining mortality among men reflects the long-term benefits of tobacco control and advances in cancer care, less favourable or increasing trends among women—particularly for lung and breast cancer—suggest delayed effects of prevention strategies and historical differences in exposure to risk factors [27,28,29]. These findings highlight the long latency between behavioural changes and measurable population-level outcomes, especially in older age groups. They also point to potential gaps in preventive interventions targeting older women, who may be underrepresented in screening programmes or experience delayed diagnoses due to atypical symptom presentation and competing comorbidities.

Beyond behavioural risk factors, the observed mortality patterns likely reflect broader systemic and demographic determinants, including population ageing, healthcare accessibility, and inequalities in preventive care. Older adults constitute a highly heterogeneous population with respect to functional status, comorbidity burden, and healthcare utilisation, which may partly explain the divergent trends observed across sex and age groups. In particular, delayed diagnosis, underutilisation of screening programmes, and lower treatment intensity among the oldest-old may contribute to persistently high or increasing mortality from selected malignancies, despite overall improvements in cancer care [30,31].

Socioeconomic factors, particularly educational attainment, represent an additional important determinant of cancer mortality trends in Poland. National data indicate pronounced educational gradients in cancer mortality, with substantially higher risk observed among individuals with primary or vocational education compared with those with tertiary education. Among men, cancer mortality in lower educational groups is over 85% higher than among those with higher education, while among women the excess risk exceeds 50% [32]. These inequalities are closely linked to differences in health-related behaviours, notably tobacco use, which remains almost three times more prevalent among individuals with lower educational attainment than among those with higher education (34% vs. 13%) [33]. Moreover, daily smoking prevalence is substantially higher in lower-income groups than in higher-income groups (25% vs. 16%) [34], contributing to socioeconomic gradients in overall cancer risk. Evidence from population-based studies further suggests that both the level of socioeconomic status and its changes over time are associated with mortality from major chronic diseases, including malignant neoplasms, although the strength and timing of these associations may vary by cancer site due to differences in latency and aetiology [35].

International evidence suggests that countries with well-integrated geriatric oncology frameworks and high coverage of age-appropriate screening programmes have achieved more favourable cancer mortality trends among older populations, especially among women. The less pronounced improvements observed in Central and Eastern Europe, including Poland, may therefore reflect structural limitations within healthcare systems—such as delayed implementation of screening programmes, workforce shortages, and uneven access to specialised oncological services—rather than differences in tumour biology alone. These findings emphasise the need for comprehensive, age-sensitive cancer control strategies that integrate prevention, early detection, and treatment with geriatric principles, particularly for the oldest age groups and for women [36,37].

Comparison of SDRs for cancer mortality revealed substantial territorial disparities across Poland. The lowest cancer mortality was consistently observed in the Podkarpackie voivodeship. This region also ranks among the leading voivodeships in terms of life expectancy for both men and women. In 2022, life expectancy at birth for women in Poland was 81.1 years, compared with 82.5 years in the Podkarpackie voivodeship, while corresponding values for men were 73.4 years nationwide and 74.7 years in the Podkarpackie voivodeship [38]. The low cancer mortality rates observed in the Podkarpackie voivodeship, particularly for lung cancer, are consistent with the lowest prevalence of smoking in Poland, which in 2022 amounted to 19.0%, compared with the national average of 22.0% [33]. Furthermore, a report by the WHO Regional Office for Europe entitled “Social inequalities in health in Poland” demonstrated that among individuals aged over 65 years, the risk of death from malignant neoplasms is higher among urban residents than among rural residents. Specifically, cancer mortality among men living in cities is approximately 5% higher than among those living in rural areas, while among women it is more than one quarter higher (27%), largely due to substantially higher mortality from lung, breast, and cervical cancers [39]. The Podkarpackie voivodeship has the lowest level of urbanisation in Poland, with only 41% of the population living in urban areas [40], which may further contribute to the relatively low cancer mortality observed in this region. These observations highlight substantial geographical differences in cancer mortality across Poland, which are partly related to regional variation in population ageing, health behaviours, and urbanisation.

### Limitations

This study has several strengths, including its nationwide scope, long observation period, and use of standardised mortality measures and joinpoint regression to identify temporal changes. However, certain limitations should be acknowledged. Mortality data based on death certificates may be subject to misclassification of causes of death, particularly in older age groups with multiple comorbidities. Moreover, the lack of individual-level data precluded adjustment for socioeconomic status, smoking history, or treatment patterns. Despite these limitations, the observed long-term trends provide robust and policy-relevant insights into the evolving cancer burden among older adults in Poland.

## 5. Conclusions

Cancer mortality trends in Poland show marked heterogeneity across cancer types and between sexes, with periods of both improvement and stagnation observed over the study period. Despite overall progress in selected cancers, recent years indicate a slowing of previously favourable trends, underscoring persistent challenges in cancer control. These findings emphasise the need for sustained prevention efforts, effective early detection, and timely access to oncological care, supported by continuous population-level monitoring to inform public health policy.

## Data Availability

Data available on request from corresponding authors.

## Supplementary Materials

The following supporting information can be downloaded at: https://www.mdpi.com/article/10.3390/cancers18030447/s1, Table S1. Standardised death rates (SDRs) due to the most common causes in women in 2000–2022. Table S2. Standardised death rates (SDRs) due to the most common causes in men in 2000–2022. Table S3. Time trends in standardised death rates (SDRs) due to the most common causes in women aged 65–74 years in 2000–2020—joinpoint regression analysis. Table S4. Standardised death rates (SDRs) due to the most common cancers among women in 2022, by voivodeship. Table S5. Standardised death rates (SDRs) due to the most common cancers among men in 2022, by voivodeship.

## Abbreviations

The following abbreviations are used in this manuscript:

SDRs	Standardised Death Rates
APC	Annual Percentage Change
AAPC	Average Annual Percentage Change

## References

[1] Ageing and Health. World Health Organization. https://www.who.int/news-room/fact-sheets/detail/ageing-and-health.

[2] Gianfredi V., Nucci D., Pennisi F., Maggi S., Veronese N., Soysal P. (2025). Aging, longevity, and healthy aging: The public health approach. Aging Clin. Exp. Res..

[3] Xi J.Y., Liang B.H., Zhang W.J., Yan B., Dong H., Chen Y.Y., Lin X., Gu J., Hao Y.T. (2025). Effects of population aging on quality of life and disease burden: A population-based study. Glob. Health Res. Policy.

[4] Wang J., Zhang Y., Wang P., Wang Y., Zhang H., Jia Z., Wei Y., Liang H. (2025). Global statistics and risk factors of neoplasms in the elderly, and the impact of aging on neoplasms from 1990 to 2021. Int. J. Surg..

[5] Sun K.X., Liang X., Zhu Q., Wu H.L., Zhang G.Y., Yao Y.F., Li X., Zheng R.S., Zuo J., Wei W.-Q. (2025). Global patterns and trends in cancer-related premature death and their impact on life expectancy across 185 countries: A population-based analysis. Mil. Med. Res..

[6] Raza S.A. (2025). Emerging Trends in Global Cancer Epidemiology. Cancers.

[7] Prathap R., Kirubha S., Rajan A.T., Manoharan S., Elumalai K. (2024). The increasing prevalence of cancer in the elderly: An investigation of epidemiological trends. Aging Med..

[8] Wojciechowska U., Didkowska J.A., Barańska K., Miklewska M., Michałek I., Olasek P., Jawołowska A. (2024). Cancer in Poland in 2022.

[9] Revision of the European Standard Population. https://ec.europa.eu/eurostat/documents/3859598/5926869/KS-RA-13-028-EN.PDF/e713fa79-1add-44e8-b23d-5e8fa09b3f8f.

[10] Kim H., Fay M.P., Feuer E.J., Midhune D.N. (2000). Permutation tests for joinpoint regression with applications to cancer rates. Stat. Med..

[11] Tse L.A., Lin X., Li W., Qiu H., Chan C.K., Wang F., Yu I.T., Leung C.C. (2018). Smoking cessation sharply reduced lung cancer mortality in a historical cohort of 3185 Chinese silicotic workers from 1981 to 2014. Br. J. Cancer.

[12] Bray F.I., Weiderpass E. (2010). Lung cancer mortality trends in 36 European countries: Secular trends and birth cohort patterns by sex and region 1970–2007. Int. J. Cancer.

[13] Moryson W., Stawinska-Witoszynska B. (2021). Premature Mortality Due to Tobacco-Related Malignancies in Poland. Int. J. Gen. Med..

[14] Moryson W., Stawinska-Witoszynska B. (2021). Excess Mortality of Males Due to Malignant Lung Cancer in OECD Countries. Int. J. Environ. Res. Public Health.

[15] Alotaibi A., Ali A., Brown C., Sherbeny F. (2022). Exploratory Analysis of Survival and Mortality Rates Among Older Lung Cancer Patients Utilizing Different Treatment Modalities. Innov. Pharm..

[16] Carioli G., Malvezzi M., Bertuccio P., Hashim D., Waxman S., Negri E., Boffetta P., La Vecchia C. (2019). Cancer mortality in the elderly in 11 countries worldwide, 1970–2015. Ann. Oncol..

[17] Didkowska J.A., Wojciechowska U., Barańska K., Miklewska M., Michałek I., Olasek P. Nowotwory Złośliwe w Polsce w 2021 Roku. https://onkologia.org.pl/sites/default/files/publications/2024-01/0_krn-2023-book-2024-01-22.pdf.

[18] European Society for Medical Oncology Death Rates from Lung Cancer Will Fall Overall in the EU and UK in 2023, but Rise Among Women in France, Italy and Spain [Annals of Oncology Press Release]. https://ecancer.org/en/news/22768-death-rates-from-lung-cancer-will-fall-overall-in-the-eu-and-uk-in-2023-but-rise-among-women-in-france-italy-and-spain.

[19] Torre L.A., Siegel R.L., Ward E.M., Jemal A. (2014). International variation in lung cancer mortality rates and trends among women. Cancer Epidemiol. Biomark. Prev..

[20] Pikala M., Krzywicka M., Burzyńska M. (2022). Excess mortality in Poland during the first and second wave of the COVID-19 pandemic in 2020. Front. Public Health.

[21] Maringe C., Spicer J., Morris M., Purushotham A., Nolte E., Sullivan R., Rachet B., Aggarwal A. (2020). The impact of the COVID-19 pandemic on cancer deaths due to delays in diagnosis in England, UK: A national, population-based, modelling study. Lancet.

[22] Lai A.G., Pasea L., Banerjee A., Hall G., Denaxas S., Hoong Chang W., Katsoulis M., Williams B., Pillay D., Noursadeghi M. (2020). Estimated impact of the COVID-19 pandemic on cancer services and excess 1-year mortality in people with cancer and multimorbidity: Near real-time data on cancer care, cancer deaths and a population-based cohort study. BMJ Open.

[23] Hanna T.P., King W.D., Thibodeau S., Jalink M., Paulin G.A., Harvey-Jones E., O’Sullivan D.E., Booth C.M., Sullivan R., Aggarwal A. (2020). Mortality due to cancer treatment delay: Systematic review and meta-analysis. BMJ.

[24] Chen R.C., Haynes K., Du S., Barron J., Katz A.J. (2021). Association of Cancer Screening Deficit in the United States with the COVID-19 Pandemic. JAMA Oncol..

[25] Ministerstwo Zdrowia (2022). Wpływ Pandemii COVID-19 na Potrzeby Zdrowotne w Polsce. Choroby Onkologiczne. Warszawa. https://basiw.mz.gov.pl/wp-content/uploads/2022/06/220330_Wplyw_pandemii_COVID-19_na_potrzeby_zdrowotne_onkologia.pdf.

[26] Cook M.B., McGlynn K.A., Devesa S.S., Freedman N.D., Anderson W.F. (2011). Sex disparities in cancer mortality and survival. Cancer Epidemiol. Biomark. Prev..

[27] Lu D.N., Jiang Y., Zhang W.C., Du R.K., Zeng A., Wu Y.M., Zhou X. (2025). Lung Cancer incidence in both sexes across global areas: Data from 1978 to 2017 and predictions up to 2035. BMC Pulm. Med..

[28] May L., Shows K., Nana-Sinkam P., Li H., Landry J.W. (2023). Sex Differences in Lung Cancer. Cancers.

[29] Bernardez B., Higuera O., Martinez-Callejo V., Cardeña-Gutiérrez A., Marcos Rodríguez J.A., Santaballa Bertrán A., Majem M., Moreno-Martínez M.E. (2025). Sex and gender differences in cancer pathogenesis and pharmacology. Clin. Transl. Oncol..

[30] Sung H., Ferlay J., Siegel R.L., Laversanne M., Soerjomataram I., Jemal A., Bray F. (2021). Global Cancer Statistics 2020: GLOBOCAN Estimates of Incidence and Mortality Worldwide for 36 Cancers in 185 Countries. CA Cancer J. Clin..

[31] Wildiers H., Heeren P., Puts M., Topinkova E., Janssen-Heijnen M.L., Extermann M., Falandry C., Artz A., Brain E., Colloca G. (2014). International Society of Geriatric Oncology consensus on geriatric assessment in older patients with cancer. J. Clin. Oncol..

[32] Wojtyniak B., Smaga A. (2025). Sytuacja Zdrowotna Ludności Polski i jej Uwarunkowania 2025.

[33] Zimny-Zając A. Narodowy Test Zdrowia Polaków. Raport 2022. Kraków 2022. https://ocdn.eu/medonet/medonet%20market/NTZP2022_raport_display.pdf?fbclid=IwAR2OFicRpr7Lw7zxYBu5yfUJUfUCXclxgz3zNHRx3ukdRV-AVxxV8FdmrRo.

[34] OECD (2023). EU Country Cancer Profile: Poland 2023; EU Country Cancer Profiles.

[35] Polak M., Genowska A., Szafraniec K., Fryc J., Jamiołkowski J., Pająk A. (2019). Area-Based Socio-Economic Inequalities in Mortality from Lung Cancer and Respiratory Diseases. Int. J. Environ. Res. Public Health.

[36] Allemani C., Matsuda T., Di Carlo V., Harewood R., Matz M., Nikšić M., Bonaventure A., Valkov M., Johnson C.J., Estève J. (2018). Global surveillance of trends in cancer survival 2000-14 (CONCORD-3): Analysis of individual records for 37 513 025 patients diagnosed with one of 18 cancers from 322 population-based registries in 71 countries. Lancet.

[37] Lakkis N.A., Mokalled N.M., Osman M.H., Musharrafieh U.M. (2025). Cancer in the Oldest-Old Population in the MENA Region: Epidemiology and Temporal Trends Based on GBD 2021. Cancer Control.

[38] Life Expectancy Tables of Poland 2022. https://stat.gov.pl/en/topics/population/life-expectancy/life-expectancy-tables-of-poland-2022,2,16.html.

[39] Social Inequalities in Health in Poland. WHO Regional Office for Europe 2012. https://iris.who.int/server/api/core/bitstreams/f626b919-2eb2-487c-9bb1-99b5b37f1ef4/content.

[40] Statistics Poland. Local Data Bank. https://bdl.stat.gov.pl/bdl/start.

