# Supplementary material for: Cancer Mortality in an Ageing Population: Evidence of Sex-Specific Divergence from a National Study in Poland
Source: Cancers (Basel). 2026 Jan 30;18(3):447. doi: 10.3390/cancers18030447 (PMC12896599; doi:10.3390/cancers18030447)
Supplement: Supplementary file 1 [file cancers-18-00447-s001.zip › cancers-4103920-supplementary.pdf]

Supplementary Table S1. Standardized death rates (SDR) due to the most common causes in women in 2000-2022

Year	Death causes											
	Malignant neoplasms (total)	Malignant neoplasm of bronchus and lungs	Malignant neoplasm of stomach	Malignant neoplasm of colorectum	Malignant neoplasm of breast	Malignant neoplasm of pancreas	Malignant neoplasms (total)	Malignant neoplasm of bronchus and lungs	Malignant neoplasm of stomach	Malignant neoplasm of colorectum	Malignant neoplasm of breast	Malignant neoplasm of pancreas
	Women aged 65-74						Women aged 75+					
2000	631.4	74.2	38.6	68.9	67.8	35.2	1114.6	89.4	85.5	152.5	112.1	65.4
2001	616.8	76.5	35.1	69.4	69.8	32.4	1133.2	99.0	86.1	152.1	116.2	67.7
2002	600.5	76.3	32.3	67.5	66.0	33.1	1130.3	99.8	82.0	155.1	111.2	67.1
2003	606.6	80.5	32.0	63.4	67.6	35.8	1139.6	100.9	78.7	156.5	114.7	68.1
2004	597.0	76.4	32.1	65.1	66.4	32.8	1127.4	99.3	74.9	155.7	109.7	62.9
2005	596.2	81.3	30.8	67.6	69.2	33.6	1131.5	100.5	66.3	153.3	117.6	63.5
2006	589.9	83.5	28.9	60.3	68.9	35.8	1113.6	101.5	66.6	150.3	118.3	66.5
2007	597.1	90.1	28.7	64.9	70.7	37.0	1112.6	106.2	67.2	148.0	116.5	68.0
2008	604.6	95.0	29.2	65.9	69.6	37.8	1099.1	103.3	68.2	156.7	117.3	71.0
2009	590.0	94.7	26.0	65.4	68.0	35.1	1071.0	108.8	58.3	157.5	113.4	64.2
2010	598.4	105.3	25.5	69.4	70.3	36.2	1033.9	107.2	57.8	153.1	110.8	62.9
2011	590.4	105.5	22.6	65.6	71.2	36.1	1004.1	105.5	54.9	146.9	109.8	59.9
2012	603.9	109.8	24.8	70.2	73.8	35.5	1025.4	107.4	52.9	153.2	113.8	66.9
2013	594.6	115.2	24.5	62.9	74.2	36.6	997.6	105.0	52.5	153.7	116.6	64.3
2014	622.1	134.2	26.1	64.4	79.8	39.1	994.3	115.9	50.6	146.7	116.9	65.4
2015	623.4	137.2	24.5	65.2	76.9	35.7	1081.3	116.1	51.4	158.3	136.6	65.2
2016	619.9	139.3	22.8	64.6	78.3	39.2	1067.5	126.3	50.6	151.1	140.1	63.3
2017	627.4	144.4	22.8	65.8	78.3	35.5	1043.0	124.1	45.7	152.5	146.3	62.0
2018	627.4	153.4	20.5	60.5	75.8	36.9	1061.2	126.4	45.6	153.8	155.4	62.5
2019	628.7	153.2	19.9	61.4	76.3	39.4	1052.4	136.7	42.7	151.5	155.2	63.6
2020	610.7	148.7	19.2	61.1	79.8	37.0	1053.6	135.6	42.0	145.0	153.4	59.6
2021	585.0	145.9	18.0	57.9	73.0	33.7	981.5	136.3	40.0	138.3	142.1	56.4
2022	580.9	144.6	16.8	59.1	70.5	35.7	1041.5	144.3	39.0	143.7	154.1	57.7

Supplementary Table S2. Standardized death rates (SDR) due to the most common causes in men in 2000-2022

Year	Death causes											
	Malignant neoplasms (total)	Malignant neoplasm of bronchus and lungs	Malignant neoplasm of stomach	Malignant neoplasm of colorectum	Malignant neoplasm of prostate	Malignant neoplasm of pancreas	Malignant neoplasms (total)	Malignant neoplasm of bronchus and lungs	Malignant neoplasm of stomach	Malignant neoplasm of colorectum	Malignant neoplasm of prostate	Malignant neoplasm of pancreas
	Men aged 65-74						Men aged 75+					
2000	1440.1	535.9	116.5	124.3	93.6	51.8	2226.5	520.9	205.3	238.3	332.2	73.9
2001	1431.3	531.7	106.5	125.5	93.1	53.1	2310.1	537.3	200.2	240.1	361.3	77.7
2002	1440.4	536.7	111.2	130.9	94.2	51.2	2364.1	562.2	199.2	254.1	373.9	81.6
2003	1406.4	512.5	105.4	127.1	82.5	55.9	2337.8	551.3	189.1	259.3	360.4	77.8
2004	1405.6	513.5	98.9	133.3	87.6	51.4	2366.3	546.0	189.3	255.3	365.5	77.9
2005	1392.0	507.0	99.5	132.9	86.7	53.8	2316.7	559.1	176.0	257.2	355.5	73.3
2006	1380.1	501.3	97.7	133.6	86.7	51.2	2319.0	551.0	166.9	283.7	359.0	83.1
2007	1374.5	492.6	96.2	139.5	89.5	55.6	2326.5	555.3	169.6	283.7	367.2	80.0
2008	1327.3	480.1	89.8	147.7	87.3	54.7	2336.0	580.6	166.4	295.0	348.0	86.5
2009	1314.7	454.0	84.9	145.9	93.1	57.1	2288.9	558.2	156.9	290.5	344.4	82.6
2010	1285.6	448.7	86.5	149.4	85.4	58.4	2202.3	543.8	155.4	295.9	326.5	77.6
2011	1223.4	421.9	82.4	143.2	84.9	51.3	2192.6	532.6	152.9	304.3	325.1	76.0
2012	1221.7	419.8	78.4	145.5	83.2	57.2	2203.1	529.2	145.5	308.9	333.5	80.9
2013	1195.8	411.1	73.6	141.4	89.4	53.6	2133.5	516.2	146.0	301.2	323.2	78.2
2014	1194.6	402.6	74.0	141.4	92.0	57.8	2104.5	519.3	142.5	307.7	318.3	81.7
2015	1212.8	404.8	74.8	147.2	94.7	55.8	2322.9	525.5	140.1	332.7	364.4	76.5
2016	1183.5	406.0	67.2	140.4	93.8	52.3	2270.0	507.4	137.9	331.9	380.6	79.7
2017	1138.9	378.1	64.3	138.4	95.7	54.2	2201.4	492.9	129.0	322.9	378.1	72.8
2018	1159.1	378.5	64.3	138.5	99.1	53.0	2198.5	494.3	126.1	326.3	380.6	79.8
2019	1106.6	357.4	62.1	135.6	92.1	52.9	2099.3	450.3	117.9	316.8	376.0	70.1
2020	1076.3	333.1	59.3	137.6	95.9	51.3	2168.7	443.3	124.2	325.0	388.9	69.3
2021	999.7	304.0	55.5	123.2	89.0	46.9	1982.2	403.7	106.3	292.5	366.4	70.2
2022	1006.0	308.7	54.3	125.3	87.9	51.2	2076.9	414.8	101.7	311.7	381.7	74.3

Supplementary Table S3. Time trends in standardized death rates (SDRs) due to the most common causes in women aged 65–74 years in 2000–2020 – joinpoint regression analysis.

Causes of death	Number of joinpoints	Years	APC (95% CI)	AAPC (95% CI)
Women aged 65-74				
Malignant neoplasms (C00-C97) including:	2	2000-2006	-1.0* (-1.7; -0.3)	-0.4* (-0.7; 0.0)
		2006-2019	0.5*(0.3; 0.7)	
		2019-2022	-2.9* (-4.9; -0.9)	
Malignant neoplasm of bronchus and lungs (C34)	2	2000-2005	1.3 (-0.7; 3.4)	3.5* (2.3; 3.8)
		2005-2018	5.2* (4.6; 5.8)	
		2018-2022	-1.7 (-4.5; 1.2)	
Malignant neoplasm of stomach (C16)	2	2000-2011	-3.8* (-4.4; -3.2)	-3.5* (-4.6; -2.3)
		2011-2014	-2.3 (-6.6; 12.0)	
		2014-2022	-5.0* (-6.0; -4.1)	
Malignant neoplasm of colorectum (C18-C20)	0	2000-2022	-0.5* (-0.8; -0.2)	
Malignant neoplasm of breast (C50)	2	2000-2009	0.3 (-0.5; 1.0)	0.3 (-0.3; 0.9)
		2009-2016	2.0* (0.7; 3.4)	
		2016-2022	-1.5* (-2.9; -0.2)	
Malignant neoplasm of pancreas (C25)	0	2000-2022	0.4* (0.1; 0.7)	
Women aged 75+				
Malignant neoplasms (C00-C97) including:	2	2000-2007	-0.1 (-1.2; 0.9)	-0.4 (-1.1; 0.4)
		2007-2011	-2.3 (-6.1; 1,6)	
		2011-2022	0.2 (-0.3; 0.8)	
Malignant neoplasm of bronchus and lungs (C34)	1	2000-2013	1.1* (0.7; 1.5)	1.9* (1.5; 2.3)
		2013-2022	3.1* (2.3; 3.8)	
Malignant neoplasm of stomach (C16)	0	2000-2022	-3.5* (-3.8; -3.3)	
Malignant neoplasm of colorectum (C18-C20)	1	2000-2018	-0.1 (-0.3; 0.2)	-0.4 (-0.8; 0.0)
		2018-2022	-2.1 (-4.3; 0.2)	
Malignant neoplasm of breast (C50)	2	2000-2013	0.0 (-0.5; 0.6)	1.2* (0.3; 2.1)
		2013-2018	6.5* (3.1; 10.0)	

		2018-2022	-1.4 (-4.5; 1.9)	
Malignant neoplasm of pancreas (C25)	0	2000-2022	-0.6* (-0.8; -0.3)	
Men aged 65-74				
Malignant neoplasms (C00-C97) including:	2	2000-2004	-0.6 (-2.2; 1.1)	-1.7* (-2.1; -1.3)
		2004-2018	-1.5* (-1.8; -1.3)	
		2018-2022	-3.3* (-4.8; -1.7)	
Malignant neoplasm of bronchus and lungs (C34)	2	2000-2005	-1.3 (-2.9; 0.4)	-2.6* (-3.2; -2.1)
		2005-2018	-2.3* (-2.8; -1.9)	
		2018-2022	-5.3* (-7.4; -3.0)	
Malignant neoplasm of stomach (C16)	0	2000-2022	-3.3* (-3.5; -3.2)	
Malignant neoplasm of colorectum (C18-C20)	1	2000-2010	1.9* (1.3; 2.6)	0.2 (-0.1; 0.6)
		2010-2022	-1.2* (-1.7; -0.7)	
Malignant neoplasm of prostate (C61)	2	2000-2012	-0.5 (-1.1; 0.1)	-0.2 (-1.1; 0.7)
		2012-2018	2.4 (-0.1; 4.9)	
		2018-2022	-3.0 (-6.3; 0.4)	
Malignant neoplasm of pancreas (C25)	1	2000-2014	0.5 (0.0; 1.1)	-0.3 (-0.8; 0.3)
		2014-2022	-1.7* (-3.0; -0.3)	
Men aged 75+				
Malignant neoplasms (C00-C97) including:	0	2000-2022	-0.5* (-0.7; -0.3)	
Malignant neoplasm of bronchus and lungs (C34)	2	2000-2008	0.7* (0.0; 1.4)	-1.3* (-1.8; -0.8)
		2008-2018	-1.5* (-2.1; -0.9)	
		2018-2022	-4.8* (-6.6; -2.9)	
Malignant neoplasm of stomach (C16)	1	2000-2020	-2.7* (-2.8; -2.5)	-3.2* (-3.8; -2.6)
		2020-2022	-8.5* (-14.3; -2.4)	
Malignant neoplasm of colorectum (C18-C20)	1	2000-2016	2.0* (1.7; 2.4)	1.1* (0.6; 1.5)
		2016-2022	-1.4 (-2.9; 0.0)	
Malignant neoplasm of prostate (C61)	3	2000-2002	6.0 (-1.5; 14.1)	0.6 (-0.6; 1.7)
		2002-2013	-1.5* (-2.0; -0.9)	
		2013-2016	5.7 (-1.8; 13.8)	
		2016-2022	0.0 (-1.3; 1.2)	
Malignant neoplasm of pancreas (C25)	1	2000-2008	1.1 (-0.3; 2.5)	-0.3 (-.0.9; 0.3)

	2008-2022	-1.0* (-1.6; -0.4)	

* $p<0,05$

Supplementary Table S4. Standardized death rates (SDRs) due to the most common cancers among women in 2022, by voivodeship.

Voivodeship	Death causes											
	Malignant neoplasms (total)	Malignant neoplasm of bronchus and lungs	Malignant neoplasm of stomach	Malignant neoplasm of colorectum	Malignant neoplasm of breast	Malignant neoplasm of pancreas	Malignant neoplasms (total)	Malignant neoplasm of bronchus and lungs	Malignant neoplasm of stomach	Malignant neoplasm of colorectum	Malignant neoplasm of breast	Malignant neoplasm of pancreas
	Women aged 65-74						Women aged 75+					
Dolnośląskie	623.5	173.7	17.0	64.9	63.7	36.9	1077.5	172.1	31.0	170.1	162.0	54.8
Kujawsko-pomorskie	662.5	196.2	14.4	63.8	85.5	28.1	1050.3	174.2	50.1	146.9	156.5	64.2
Lubelskie	530.8	122.1	10.7	53.6	70.4	29.9	847.6	96.0	33.5	120.1	120.1	48.9
Lubuskie	590.0	153.9	17.1	63.5	63.2	50.9	954.3	188.2	46.6	129.1	135.9	38.7
Łódzkie	612.6	150.7	14.2	64.2	80.1	42.9	955.4	134.4	36.1	118.2	146.6	54.2
Małopolskie	562.1	125.9	15.4	49.4	73.0	37.6	1162.9	130.4	39.4	147.3	173.3	64.1
Mazowieckie	597.2	152.4	16.9	54.4	76.9	35.9	1014.4	142.8	38.8	132.6	152.6	52.6
Opolskie	553.7	110.2	15.5	73.4	60.1	31.1	1070.4	106.3	59.5	150.7	153.1	51.2
Podkarpackie	441.6	87.9	14.1	48.4	65.7	32.0	829.1	78.5	37.8	116.2	101.9	54.9
Podlaskie	479.4	114.5	17.3	44.0	55.0	33.1	961.2	104.7	17.9	155.6	152.3	36.1
Pomorskie	675.2	192.4	24.8	65.2	70.7	53.3	1132.4	218.8	40.3	148.3	147.9	69.0
Śląskie	639.0	137.5	13.9	70.9	85.9	38.8	1046.5	124.2	43.5	145.9	164.9	60.6
Świętokrzyskie	517.7	114.8	15.1	45.8	47.5	39.5	1054.0	84.4	41.3	132.6	157.9	43.4
Warmińsko-mazurskie	654.7	194.1	26.5	70.1	56.3	23.0	964.5	135.7	28.9	162.9	139.6	47.2
Wielkopolskie	583.2	128.8	18.3	63.9	79.6	34.0	1025.4	153.7	38.3	151.2	164.2	57.2
Zachodniopomorskie	584.9	154.0	21.5	66.5	69.1	33.4	969.3	153.2	40.3	151.2	119.0	65.2

Supplementary Table S5. Standardized death rates (SDRs) due to the most common cancers among men in 2022, by voivodeship.

Voivodeship	Malignant neoplasms (total)	Malignant neoplasm of bronchus and lungs	Malignant neoplasm of stomach	Malignant neoplasm of colorectum	Malignant neoplasm of prostate	Malignant neoplasm of pancreas	Malignant neoplasms (total)	Malignant neoplasm of bronchus and lungs	Malignant neoplasm of stomach	Malignant neoplasm of colorectum	Malignant neoplasm of prostate	Malignant neoplasm of pancreas
	Men aged 65-74						Men aged 75+					
Dolnośląskie	1053.9	326.2	54.3	120.2	84.8	64.2	2183.9	459.0	109.0	340.2	413.4	89.8
Kujawsko-pomorskie	1104.3	331.2	47.6	161.2	92.7	46.1	2029.4	441.0	94.2	330.1	364.1	85.2
Lubelskie	995.7	304.7	42.8	128.5	88.7	49.7	1816.0	351.2	60.7	246.8	350.6	59.2
Lubuskie	950.5	297.3	38.7	134.2	86.2	58.9	1698.3	396.4	75.7	228.6	283.0	66.6
Łódzkie	1011.9	312.4	50.0	131.9	93.4	50.6	1877.9	388.3	102.6	251.1	349.9	57.6
Małopolskie	1032.2	304.9	69.3	104.8	89.4	56.4	2287.9	410.6	113.0	308.2	429.1	72.8
Mazowieckie	1005.5	334.9	43.4	120.6	87.1	53.6	1961.2	387.6	91.7	306.4	371.9	85.5
Opolskie	1032.7	302.2	49.5	139.3	81.1	47.1	2132.5	376.3	111.5	371.9	341.6	84.9
Podkarpackie	875.0	233.2	61.3	117.1	90.5	47.3	1627.2	275.4	84.6	226.1	334.2	57.0
Podlaskie	917.3	290.6	37.0	122.2	93.8	45.9	1883.6	297.0	114.1	292.8	420.1	40.8
Pomorskie	1127.0	330.9	60.3	142.2	100.7	57.8	2194.0	524.2	113.5	317.1	371.9	66.6
Śląskie	1055.1	295.9	62.4	151.1	94.5	48.4	1956.0	356.7	104.2	331.2	365.7	64.2
Świętokrzyskie	983.5	280.9	55.7	102.9	106.6	50.8	2239.2	353.2	98.7	309.2	460.5	69.7
Warmińsko-mazurskie	1025.9	328.7	61.9	119.4	118.3	49.2	2093.9	469.8	110.2	332.2	334.9	58.8
Wielkopolskie	1030.7	325.1	58.1	133.2	88.3	56.0	2024.5	412.4	100.7	296.1	365.6	71.7
Zachodniopomorskie	1047.9	321.6	64.2	136.2	80.1	55.2	2105.2	502.1	103.4	277.1	389.6	53.0
